# Microglia activation due to obesity programs metabolic failure leading to type two diabetes

**DOI:** 10.1038/nutd.2017.10

**Published:** 2017-03-20

**Authors:** R Maldonado-Ruiz, L Montalvo-Martínez, L Fuentes-Mera, A Camacho

**Affiliations:** 1 Neuroscience Unit, Center for Research and Development in Health Sciences, Autonomous University of Nuevo Leon, Leon, Mexico; 2 Laboratory of Virology and Immunology, Faculty of Life Sciences, Autonomous University of Nuevo Leon, Leon, Mexico; 3 Department of Biochemistry, Faculty of Medicine, Autonomous University of Nuevo Leon, Leon, Mexico

## Abstract

Obesity is an energy metabolism disorder that increases susceptibility to the development of metabolic diseases. Recently, it has been described that obese subjects have a phenotype of chronic inflammation in organs that are metabolically relevant for glucose homeostasis and energy. Altered expression of immune system molecules such as interleukins IL-1, IL-6, IL-18, tumor necrosis factor alpha (TNF-α), serum amyloid A (SAA), and plasminogen activator inhibitor-1 (PAI-1), among others, has been associated with the development of chronic inflammation in obesity. Chronic inflammation modulates the development of metabolic-related comorbidities like metabolic syndrome (insulin resistance, glucose tolerance, hypertension and hyperlipidemia). Recent evidence suggests that microglia activation in the central nervous system (CNS) is a priority in the deregulation of energy homeostasis and promotes increased glucose levels. This review will cover the most significant advances that explore the molecular signals during microglia activation and inflammatory stage in the brain in the context of obesity, and its influence on the development of metabolic syndrome and type two diabetes.

## Introduction

Obesity is an important risk factor for the morbidity and mortality associated with metabolic syndrome, type 2 diabetes mellitus, hypertension, dyslipidemia, cancer, neurodegenerative diseases and other non-communicable chronic diseases.^1^ In 2014, more than 1.9 billion adults were overweight with a body mass index (BMI) greater or equal to 25 kg m^−2^ and over 600 million were obese with a BMI greater or equal to 30 kg m^−2^. In children under age 5, 42 million worldwide were obese.^2^ It has been proposed that increase in obesity is related to global trade liberalization, economic growth and rapid urbanization, changing diets and population lifestyles. Developing countries are the most affected by obesity and are predicted to continue to be so in the next years.^2^ Statistical analysis predicts that by 2025, global obesity will reach a prevalence of 18% in men and 21% in women.^2^ Therefore, the study of molecular mechanisms involved in obesity is fundamental to tackle this major public health problem.

Development of obesity requires a positive energy balance. However, to date, it is not entirely clear why the expansion of adipose tissue in obese individuals is strongly associated with insulin resistance and diabetes.^3^ There are two main hypotheses that try to explain this relationship. On one hand, this mechanism could be the result of a change in the proportion of adipokines, promoting insulin resistance.^3, 4^ The second hypothesis suggests that obesity promotes a failure of adipose tissue expansion and function, resulting in the release of fatty acids, and their transport and accumulation in peripheral organs such as skeletal muscle, liver, beta cells or myocardium, a process known as **lipotoxicity**.^3, 4^ In this sense, the laboratory of Professor Vidal-Puig and others have shown that the type of lipid differs in its degree of toxicity, suggesting that certain species such as ceramides and diacylglycerols promote more toxic effects.^1, 5^ As an example, an increase of up to 80% of free fatty acids (495 vs 887 μm), 112% in triglycerides (86 vs 188 mg dl^−1^), 38% in C18:0 (0.38 vs 0.26 μm) and 20% in C24:1 (0.52 vs 0.43 μm) ceramides in plasma has been demonstrated in obese humans versus lean humans.^5, 6^ The data also show increases of 76 and 83% in C16:0 (1 vs 4 μm) and C20:0 (0.1 vs 0.65 μm) ceramides in the muscle of obese subjects when compared with thin humans, respectively.^5, 6, 7^ Of interest, a 50% reduction in two phospholipids (phosphoethanolamine PE 16:0/20:4 and 18:1/20:4), and of 75% of ether lipids that contain unsaturated fatty acids (0.06–0.03 mm and 0.03–0.01 mm) in adipose tissue of morbidly obese identical twins, respectively, was demonstrated.^8^ The alterations in the lipid profile in obese humans can be reproduced in animal models with genetic obesity (ob/ob) or in obesity induced by a high-fat diet.^9^ In a second study, genetic obesity increased triacylglycerols levels by 40% (values of 18 mm) and long-chain fatty acids by 25% (values 1 μm) (11). Meanwhile, a high-fat diet in mice generated an increase of ~284% in ceramide C20 (values of 0.026 μm), and of C14, C160, C18 (values of 0.26, 0.08, 0.04 μm, respectively) in plasma.^10^ In this same model, an increase of 100% in triglyceride levels (values of 20 mM) was observed, long-chain fatty acids (values of 5 μm), ceramides (values 140 μM), and diacylglycerol (600 μm) in muscle.^10^ Overall, obesity in humans promotes increased triacylglycerol, ceramide and diacylglycerol levels that can be reproduced using animal models with genetic or nutritionally induced obesity.

## Lipotoxicity is a pathogenic mechanism in key organs for metabolic syndrome

The epidemiological data confirm a strong relationship between the increase in the degree of obesity and the development of diabetes, indicating that for every kilogram of weight gained, at the population level, there is a linear increase in the rate of diabetes.^11^ There is experimental evidence in humans and animal models that lipotoxicity, associated with increased lipids in obesity, may cause alterations in pancreatic beta cell function, and in skeletal and cardiac muscle, and can even induce hepatic steatosis, contributing to the generation of insulin resistance and type 2 diabetes.^12, 13^ Meanwhile, lipotoxicity may also occur in the CNS, as has been observed in several neurodegenerative diseases.^14, 15^ However, there is still not enough evidence to confirm the toxic damage caused by lipids to the CNS and their participation in metabolic complications associated with insulin resistance and type 2 diabetes. Thus, here we will review new evidence, suggesting the role of inflammation in the central nervous system during lipid accumulation on insulin sensitivity leading to metabolic failure.

## The role of lipotoxicity in the activation of inflammation and metabolic failure

The immune response is seen as a defense mechanism against infection; however, recent evidence has shown a strong relationship between inflammation and metabolic disorders. It is known that the immune response is involved in the balance and maintenance of an adequate metabolic state; however, it can have adverse effects under conditions of altered metabolic demand.^16^ The metabolic imbalance can lead to overactivation of the immune system, triggering a process of chronic inflammation, which has been observed in obesity and metabolic syndrome.^16, 17^ The first evidence demonstrating the presence of an inflammatory mechanism in diabetes was the pioneering discovery of the Randle glucose-fatty acid cycle,^18^ who at the time proposed as the first mechanism, the development of insulin resistance induced by fatty acids. This finding led to the discoveries of the obesity gene (ob) and a number of hormones derived from adipocytes such as leptin, adiponectin, and resistin, and various pro- and anti-inflammatory molecules termed adipokines, which redefined the endocrine and metabolic function of adipose tissue.^19^ These molecules have been shown to have autocrine/paracrine effects locally as well as an endocrine effect systemically, allowing them to act at the metabolically relevant organs, including the brain, liver, muscle, adipose tissue and pancreas.

It is well established that the increase in lipid concentrations in plasma and their accumulation in select organs and adipose tissue promotes inflammation and metabolic degeneration.^19^ However, it is not fully elucidated which are the organs or systems responsible for the inflammatory process during lipotoxicity. In this context, some years ago, we demonstrated that adipose tissue of mice exposed to high-fat diet showed macrophage recruitment with a proinflammatory profile and that this phenotype could be reversed with PPAR gamma receptor activators, indicating active lipogenesis in macrophages.^20^ With this, it is proposed that during the development of obesity there is adipocyte hypertrophy as a result of an increase in lipid-fat content and the presence of activation of a proinflammatory response that could well be extended to metabolically relevant organs.^19^ The molecular mechanism of this activation promotes a failure in the regulation of metabolic homeostasis, including intake and satiety, and the synthesis and degradation of lipids and glucose, and so on. To date, the presence of inflammation in various metabolically relevant organs has been reported during increased lipids, including skeletal muscle, liver, heart and pancreatic β-cells, which overall contributes to the pathophysiology of insulin resistance, fatty liver disease, impaired function of the myocardium and β-cell insufficiency, respectively.

## Obesity and metabolic syndrome, a chronic inflammatory process induced by an imbalance in adipose tissue homeostasis and infiltrating immune cells

Originally, adipose tissue was considered simply as an organ at rest that functioned as a reservoir in the form of triglycerides (TGA) during over nutrition. During calorie restriction, lipase-dependent adenylate cyclase (cAMP) hydrolyzes triglycerides and releases free fatty acids (FFA) to meet the energy demands of the body.^19^ Research over the past two decades has revealed that adipose tissue is a remarkably complex endocrine organ that serves as a 'master regulator' of systemic energy homeostasis.^19^ Recent evidence has shown that fat cells communicate with the rest of the body through the integration of nutritional and hormonal signals, and by secreting a number of factors known as adipokines. In recent years, great advances have been generated in the identification of adipokines and their functions, suggesting that the role of adipose tissue is a priority in the development of systemic inflammation. The importance of adipose tissue in promoting inflammation in obesity and the metabolic syndrome is evidenced by the presence of an inflammatory profile that correlates with macrophage infiltration in adipose tissue of mice and obese humans with insulin resistance.^21, 22^ The first evidence that linked the inflammatory process with obesity and metabolic syndrome was the overexpression of tumor necrosis factor alpha (TNF-α) in adipose tissue in obesity models in mice and rats, and in adipose tissue and muscle in obese humans.^23^ Subsequently, was showed a positive correlation between the degree of obesity and hyperinsulinemia, and TNF-α levels and mRNA in adipose tissue.^24^ Additionally, molecular markers of inflammation have been identified, including IL-1β, IL-6, IL-8, chemokine ligand 2 (CCL2), CCL3, CCL5, adiponectin and acute-phase markers such as C-reactive protein, serum amyloid A and fibrinogen.^25^ All these molecules coordinate inflammatory events in metabolically active organs, such as the liver, skeletal muscle and adipose tissue, which together contribute to the metabolic dysregulation and inflammation observed in obesity and metabolic syndrome. The role that each of these markers has in the development of inflammation and metabolic damage are described below.

### Tumor necrosis factor alpha (TNF-α)

TNF-α is a proinflammatory cytokine produced by a wide variety of cells, mainly macrophages and lymphocytes. In turn, in the endothelium and smooth muscle, TNF-α promotes the expression of proinflammatory genes that are dependent on a transcription factor called nuclear factor kappa-B (NF-kB). NF-kB is a process mediated by activation of the c-Jun N-terminal kinase (JNK), protein kinase C (PKC), and kinase subunit beta factor kappa-B inhibitor (IKKβ).^23, 26, 27, 28^ Meanwhile, at the final stage, these proteins stimulate transcription of the TNF-α gene, promoting positive feedback to the inflammatory cascade.

In conditions of obesity, the increase in plasma levels of TNF-α activates the PKC, IKKβ and JNK kinases, which consequently phosphorylate serine residues of the protein substrate of the insulin receptor (IRS), blocking downstream activation of the pathway, a molecular pathological event known as insulin resistance.^29^ It is known that TNF-α production originates from resident macrophages in adipose tissue.^27^ TNF-α is a key molecule in the activation of the inflammatory cascade that promotes expression of molecular targets that are downstream from its receptor, among which the increase in NF-κB expression is notable. It is known that, in obese individual, macrophages are in a proinflammatory M1 state and show increased expression of NF-κB, a decrease of the inhibitor of NF-κB (IκB) inhibitor and consequently an increase in gene transcription of the inflammatory response.^30^

### Interleukin-6 (IL-6)

Interleukin-6 is a cytokine produced by many cell types such as fibroblasts, endothelial cells, monocytes and adipocytes. In adipose tissue, production of IL-6, like TNF-α, is higher in visceral adipose tissue in obesity and metabolic syndrome.^31^ One of the main actions of IL-6, is control of the liver's production of inflammatory molecules such as C-reactive protein (CRP), whose circulating levels are a risk marker for cardiovascular events. There is a strong correlation between the protein content of IL-6 in adipose tissue and circulating levels of CRP.^32^ In addition, IL-6 plays a central role in the relationship between obesity, inflammation, and coronary heart disease; in fact, intra- abnominal visceral adipose tissue can produce greater amounts of IL-6 than subcutaneous adipose tissue,^32^ proving that central obesity increases cardiovascular risk in humans. Moreover, the production of IL-6 by adipose tissue could directly affect liver metabolism by inducing secretion of VLDL and hypertriglyceridemia, through the portal venous system.^32^ Similarly, the action of this cytokine inhibits phosphorylation of the substrate of insulin receptor 1 and 2 (IRS 1 & IRS2) and blocks the synthesis of hepatic glycogen which results in insulin resistance.

### C-reactive protein (CRP)

CRP is an acute-phase inflammatory protein used as a marker in clinical practice to assess systemic inflammation, especially *in situations* of infection. In addition, it has also been seen as a risk marker for cardiovascular disease.^33^ This protein has been shown to be an enhancer of inflammation in ischemic myocardium by local complement activation, leading to the development of myocardial infarctions and the length of these.^34^ Recently, a positive correlation between the concentrations of CRP and body mass index (BMI)^35^ has been found, in addition to IL-6 levels in adipose tissue.^32^

### Interleukin 1 beta (IL-1β)

IL-1β is a major proinflammatory cytokine, mainly produced by macrophages. It has been found that this cytokine is increased in adipose tissue, where its release has been greater in obesity.^36^ Normally, insulin action requires activation of the substrate of insulin receptor 1 (IRS1), a component that is essential for activating PI3K in response to insulin; this leads to phosphorylation of protein kinase B (also known as AKT), which results in an increase of GLUT4 transporters in the plasma membrane that maintain glucose homeostasis. However, it has been reported that overexpression of IL-1β in primary human adipocytes and in 3T3-L1 mice adipocytes causes decreased expression of the proteins: IRS1, p85 PI3K, AKT and glucose transporter GLUT4. The consequence of this effect is suppression of the signal transduction pathway of insulin, affecting glucose transport and fatty acid oxidation. Additionally, this interleukin can also act on pancreatic beta cells, inducing apoptosis through the activation of NF-κB. Moreover, blockage of IL-1β production reduces hyperglycemia and tissue inflammation.^36, 37^ In a recent study, it was demonstrated that a high content of fatty acids increases the production and secretion of IL-1β, IL-6 and IL-8 via activation of the inflammasomes NLRP3,^38^ and induces the development of obesity and insulin resistance. This amplification of cell damage is associated with the production of IL-1β, and activates the expression of more than 30 cytokines and chemokines, including CSF3, CXCL1, CXCL2, CXCL12, MCP-1, IL-8, IP-10, MIP-1α and MCP-4.^39^ In conclusion, the diverse action of IL-1β promotes the development of obesity and insulin resistance.

### Interleukin-8 (IL-8)

Serum levels of IL-8 correlate with liver inflammation and degree of nonalcoholic fatty liver disease resulting from the accumulation of free fatty acids in that organ as a consequence of obesity.^40^

### Inflammasome NLPR3

Inflammasomes are cytoplasmic multiprotein complexes that are part of the components of innate immunity, which is the first line of adaptive defense. The inflammasome that has been best characterized to date is NLRP3, whose activation induces conformational changes that allow recruitment of the adapter protein ASC, which in turn interacts with inactive procaspase-1, through the CARD domain, present in both proteins. Finally, activated caspase-1 is responsible for the maturation of pro-IL-1β and pro-IL-18, to obtain the biologically active forms, IL-1β and IL-18, and more recently, IL-6 and IL -8.^40, 41^

Its relationship with obesity emerges from a recent study, which demostrated that obesity induces the assembly of inflammasome NLRP3 by macrophages in adipose tissue, which mediates insulin resistance and early type 2 diabetes as mentioned before.^42^ This may be due to the presence of lipid molecule ceramide, which is generated by macrophages from free fatty acids.^42^ In mice with a free feed normal diet, it was found that increased expression of both NLRP3 and IL-1β in visceral adipose tissue correlates directly with body weight and adiposity compared to mice fed with a low-calorie diet. These observations have also been seen in obese humans, in whom weight loss is associated with decreased NLRP3 and IL-1β expression in subcutaneous adipose tissue. Moreover, direct participation of NLRP3 in obesity has been confirmed in studies that have shown that in gene-deficient mice fed with a high-fat diet, activation of caspase-1 and expression of pro-IL-1b in adipose tissue, and serum loss of IL-18 compared to the wild-type phenotype is reduced. In addition, it was observed that mice deficient in NLRP3 and caspase-1, and fed a high-fat diet, are more protected against insulin resistance.^42^

### Adiponectin

Adiponectin is a 30 kDa protein that shares structural similarity with collagen and TNF-α, and in which an anti-inflammatory effect has been seen in endothelial cells that is capable of inhibiting the proliferation of vascular smooth muscle cells, and suppressing conversion of macrophages to foam cells.^43^ It is highly expressed in adipose tissue and is present in the bloodstream in levels that average 5–10 mg ml^−1^ in humans. In obese subjects and patients with coronary artery disease, plasma adiponectin levels are significantly below average and inversely related to body mass index (BMI).^44^ This inverse relationship seems to be much greater with respect to visceral fat in comparison with subcutaneous peripheral fat. Co-cultures with visceral fat inhibit adiponectin secretion by subcutaneous adipose tissue, suggesting that visceral fat may secrete an inhibitor of adiponectin synthesis or secretion.^45^ In this regard, some studies have found that TNF-α exerts strong inhibition of promoter activity of adiponectin,^46^ which could explain the inverse relationship between visceral fat and circulating adiponectin levels. This suggest an obvious relationship that exists between obesity and low levels of adiponectin in plasma, other research has linked low levels of adiponectin with insulin resistance and type 2 diabetes.

Under basal conditions, adiponectin decreases insulin resistance by increasing fatty acid oxidation, which reduces triglyceride (TG) content in the muscle and liver. However, during obesity, increased lipid reserves in insulin target tissues, such as muscle and liver, are generated, leading to insulin resistance.^47^ This, as a result of adipocyte hypertrophy and alteration of its adipokine secretory profile, among which TNF-α and macrophage infiltration in adipose tissue produce these and other major interleukins (Interleukin-6 IL-6), for the development of inflammation and metabolic failure. This is again confirmed when the reduction in macrophage activity and TNF-α production *in vitro* is observed when treated with adiponectin, by inhibiting the signaling pathway of NF-κB.^46^

### CC chemokines (CCL2, CCL3, CCL5)

Chemokines are small proteins that originally were demonstrated to direct movement of circulating leukocytes to sites of inflammation or injury by chemotaxis and also that they were capable of producing inflammatory mediators. CCL2 is a chemokine that belongs to the family of CC chemokines, in which two cysteine residues are adjacent to each other.^48^ This chemokine has been associated with macrophage infiltration in adipose tissue of obese mice models and in humans with obesity.^49^ In addition to CCL2, it is known that there is overexpression of other chemokines and their receptors (CCR2, CCR3, CCR5), in adipose tissue subculture of obese subjects.^48^ Macrophage inflammatory protein-related protein-2 (MRP-2), a member of the family of CC chemokines was one of the first potent chemoattractants for monocytes, lymphocytes, neutrophils and eosinophils. It was found that MRP-2 levels were increased in mice that were fed high-fat diets as well as in patients with human atherosclerosis,^48^ indicating that MRP-2 could be associated with the pathological inflammatory process related to obesity. Moreover, it has been reported that the macrophage inflammatory protein-1 alpha (MIP-1α or CCL3) and the protein CCL5, which also belongs to the chemokines, influence adipocyte function. In particular, they function as macrophage recruitment factors, and may be a crucial link between chemokines, adipose tissue inflammation and insulin resistance.^48, 49^ Both chemokines alter lipid accumulation and leptin secretion by adipocytes. In fact, it has been demonstrated that the gene expression of CCL5, CCL7, CCL8 and CCL11 increases in visceral adipose tissue of subjects.^49^ Taken together, these chemokines are expressed in visceral adipose tissue and adipose tissue subcultures of obese subjects, playing an important role in promoting inflammation of adipose tissue in obesity.

### Serum amyloid A (SAA)

SAA is an inflammatory protein codified by the SAA1 and SAA2 genes that appear to be expressed in a coordinated manner. Its transcription can be induced by glucocorticoids and various cytokines such as TNF-α, IL-1α, IL-1β and IL-6.^50, 51^ It has been regarded as an autocrine amplifier of the proinflammatory environment because it stimulates the expression of the same cytokines that activate it (IL-6, IL-8, and CXCL1).^52^ SAA is expressed in adipose tissue, and its expression is increased greatly in obesity; circulating levels of SAA show a positive correlation with insulin resistance in obesity and type 2 diabetes.^53, 54^ Furthermore, SAA induces the production of IL-8 through the formyl peptide receptor, FPRL1, and also activates NF-κB. The same signaling pathway has been demonstrated as an important mediator of inflammation associated with insulin resistance.

### Fibrinogen

Fibrinogen is a dimeric glycoprotein found in plasma and in the alpha granules of platelets. It is a precursor of fibrin, whose importance lies in the formation of blood clots in the presence of wounds. In pathological conditions, it can result in the formation of obstructive thrombi in the circulation, increasing the risk of cardiovascular disorders, especially in obese subjects.^54^

## Central inflammation and microglia activation modulate pathological neuronal pruning and cognitive deficits

The role of neuroinflammation and pathological microglia activation on neuronal function, development and behavior have been focused on intensive research in the last years. Elegant studies have shown that microglia coordinates pruning of synapses during development shaping proper neuronal circuit structure and synaptic function.^55^ Under non pathological scenario, microglia infiltration into the brain during development leads to synaptic pruning and brain maturation. Synaptic pruning is supported by the classical complement cascade, a key component of innate immune pathogen defense, where microglia suffers changes in morphology that correlate with its phagocytic activity and receptor recognition. Proper synaptic pruning requires the CR3 ligand complement 3 interactions. CR3 is a human cell surface receptor, which consist of a differentiation molecule 11b (CD11b) and CD18, and is found on polymorphonuclear leukocytes, NK cells and mononuclear phagocytes like macrophages and microglia cells.^56, 57^ Recent evidence has shown that primary deficit in microglia such as deficient of complement receptor 3 (CR3) was sufficient to induce some autism-related behavioral and functional connectivity deficits.^58^ Also, pathological CR3-dependent synaptic pruning might occur during inflammatory neuropathologies such as stroke, trauma and neurodegenerative diseases. Under this scenario, microglial CR3 triggers long-term synaptic depression, which requires activation of nicotinamide adenine dinucleotide phosphate oxidase (NADPH oxidase), and GluA2-mediated A-amino-3-hydroxy-5-methyl-4-isoxazolepropionic acid receptor (AMPAR) internalization.^59^ Cognitive impairment and behavioral alterations related to microglia activation and synaptic pruning has also been reported during aging. Mouse deficient of progranulin gene, which is a genetic cause of frontotemporal dementia, displays profound microglia infiltration and synapses loss in the ventral thalamus, which correlates with hyperexcitability and grooming behaviors.^60^ Microglia activation and central inflammation might also be target of an external stimulus such as a virus. Synaptic elimination by microglia CR3 receptor-dependent has been reported in a mouse model infected with West Nile virus, leading to cognitive dysfunction and neurocognitive impairment.^61^ Finally, a transcriptomic study analyzing the Alzheimer's Disease Risk Genes support the role of inflammation as a leading cause of the pathology showing immune response genes (CLU, CR1, ABCA7, MS4A, CD33 and EPHA1).^62^ These evidences suggest that microglia CR3-dependent synaptic pruning during development is a physiological pathway shaping efficient neuronal circuit structure and synaptic function. However, pathological microglia CR3-dependent synaptic pruning may also occur by internal (see below) as external stimuli, leading to failure in neuronal excitability and memory and behavioral alterations. In any case, it remains to be address if microglia activation is a primary cause or a secondary consequence of neuronal deficits in humans and if it is exacerbated during lipotoxic insult such as obesity or metabolic syndrome. We will address this question below.

## Inflammation of the nervous system during obesity is key in the generation of metabolic damage and diabetes

Substantial evidence has shown that chronic inflammation in obesity is not limited to adipose tissue, but invades borders that are farther away, and can be detected in the central nervous system. This inflammatory response can originate by the recruitment of peripheral immune cells, mainly macrophages and B cells to the SNC, which promotes the generation of inflammation.^63^ The link between glucose intolerance and central inflammation was reported by Kumar M (2014), showing that in the genetic diabetic model (db/db mice) displays increase in mRNA and protein levels of key chemokines such as CXCL10, CXCL1, CCL2, CCL5, CCL3 and G-CSF, and cytokines such as IL-1β, TNF, IL-6, IFNγ and IL-1α compared to WT mice, suggesting that metabolic compromise correlate with central immune response activation despite hypercaloric state.^64^ Also, initial research by Velloso's group reported that specific low-grade chronic inflammation modulates body glucose homeostasis, supporting that hypothalamic TNFα administration to rats leads to hyperinsulinemia and insulin resistance in liver and skeletal muscle,^65^and also generates pancreas dysfunction.^66, 67^ Of note, these effect are dependent on parasympathetic signals delivered by the vagus nerve to pancreas or liver.^68, 69^ Anatomically, hypothalamus modulates plasma glucose by vagal innervation to liver, which activates the gluconeogenesis program. Reports have shown that efficient insulin signaling in mediobasal hypothalamus blocks gluconeogenesis.^70, 71^ In this context, vagal innervation seems to regulate pancreas and liver inflammation given that nerve stimulation reduces intestinal inflammation in the gut, which depend on the α7 nicotinic acetylcholine receptor.^72^ This suggest that compromising vagal innervation/stimulation to pancreas and liver might lead to inflammation; however, it is also possible that central inflammation during obesity compromises vagal innervation/stimulation, promoting organ dysfunction and plasma glucose increase.

The effect of peripheral recruitment of inflammatory inducers has been reported during lipotoxic insult induced by high-fat diet intake in mice. There is evidence of B lymphocytes recruitment into the CNS leading to the production of a pathogenic IgG antibody, which increases inflammatory cytokines and promotes polarization of M2 macrophages to the proinflammatory phenotype M1, resulting in the generation of insulin resistance.^73^ Additionally, recent studies have demonstrated that ingestion of a high saturated fat diet increases expression of inflammatory cytokines in the hypothalamus without evidence of peripheral inflammation, presumably regulated by microglia.^74, 75^ Also, recent evidence shows that high-fat diet feeding during 12–20 weeks promote increase in plasma glucose levels, which correlates with microglia activation in hypothalamus.^76, 77^ The susceptibility of CNS to inflammation during lipotoxicity is evidenced by a recent work showing that 3 days of exposure to a high-fat diet is sufficient to promote gliosis and inflammation in the hypothalamus of rats, with no apparent changes in peripheral organs.^78^ In fact, microglia activation profile in paraventricular nucleus of hypothalamus might be even programed during neonatal stage exposed to high-fat diet and persist into adulthood.^79^ While the molecular mechanism, which modulates recruitment of bone marrow-derived monocytic cells to the hypothalamus has not been descibed enterely, it has been reported that the chemokine CX3CL1 (fractalkine) is expresed in mice hypothalamus by high-fat diet exposed, leading to bone marrow-derived monocytic cells accumulates and promoting obesity and glucose intolerance.^79^ These evidences indicate that hypothalamus seems to be an early target of inflammation during positive energy balance and an active node regulating the cross-talk between CNS to liver or pancreas.

## The TLR-IKKβ-NF-κB pathway in the development of type 2 diabetes

**Figure 1:**
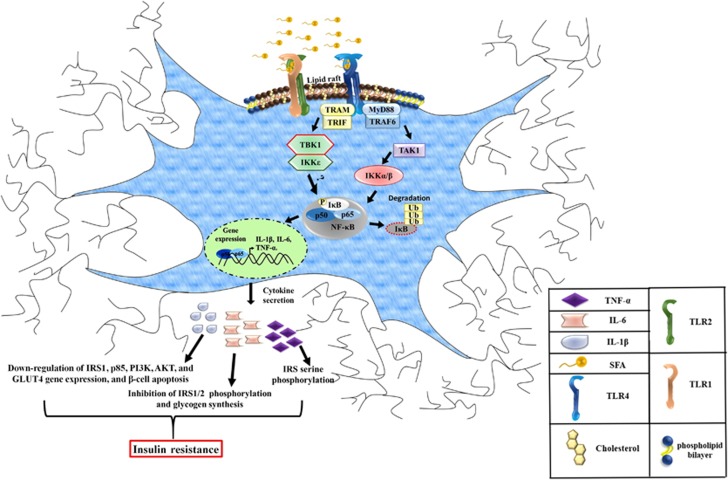
Microglia cytokine secretion during hyperlipidemia. Excess of saturated fatty acids (SFA) induce heterodimerization of toll-like receptor 1 (TLR1) with TLR2, as well as the homodimerization of TLR4, in this scenario, the adaptor protein complex MyD88-TRAF6 will be recruited to the TLR's intracellular domains, leading to TAK1 activation, and IKK (IKKα/β) downstream complex stimulation. IKKα/β dimer phosphorylates the inhibitory subunit of NF-κB, IκB, allowing the nuclear translocation of NF-κB subunits, p50 and p65, and proinflammatory cytokine gene expression, TNF-α, IL-1β and IL-6. Likewise, we proposed that, the activation of TBK1/IKKɛ, non-canonical IKKs, through the TRAM-TRIF complex, induce the phosphorylation of IκB and the nuclear translocation of p50 and p65, increasing proinflammatory cytokine gene expression in metabolic brain regions, including the hypothalamus, resulting in metabolic alterations, leading to insulin resistance.

For some years, it has been known that members of the IKK/NF-κB family have constitutive activity in the hypothalamus and can be in turn activated by different effectors, including molecular patterns associated with damage (DAMPs), molecular patterns associated with pathogens (PAMPs), cytokines, chemokines, neurotransmitters and so on, and that they modulate events of synaptic plasticity, neurotransmission and neuroprotection, and in the proliferation of neural stem cells.^80^ However, aberrant activation of the IKK/NF-κB axis has been linked to selective neuroinflammation and apoptosis independent of neurodegeneraion.^81^ In the context of metabolic damage, it has been shown that inhibition of the IKK/NF-κB axis using the terpenoid (Rb1) reduces inflammation and leptin resistance in the hypothalamus.^82^ Furthermore, it has also been shown that lipotoxicity activates the proinflammatory axis IKKβ-NF-κB in the hypothalamus, promoting the generation of insulin resistance.^83, 84, 85^ In this regard, in an elegant study Li J *et al.* demonstrated that activation of the IKKβ-NF-κB pathway during obesity, decreases neurogenesis, increases cognitive deterioration and degeneration of hypothalamic stem cells.^86, 87^ The deterioration in neural proliferation and differentiation, observed in obese mice, seems to be the result of excessive release of inflammatory cytokines such as TNF-α and IL-1β, which occurs after the activation of NF-κB, and it is also known that the IKKβ/NF-κB axis is efficiently activated by positive feedback.^86, 87^ With this, it seems that the IKKβ/NF-κB axis is a negative modulator of metabolism and is associated with cognitive impairment (see Figure 1).

The molecular mechanisms linked to the activation of CNS inflammation in conditions of lipotoxicity and its role on diabetes susceptibility are not entirely clear. However, it has been proposed that saturated fatty acids (SFA) are able to induce heterodimerization of Toll-like receptors 1 and 2 (TLR1 and TLR2), and homodimerization of Toll-like receptor 4 (TLR4), and simultaneous movement into the region of lipidic rafts, possibly by interaction of SFA with hydrophobic pockets present in the extracellular region of TLR's.^88, 89, 90^ This mechanism, promotes the expression of inflammatory cytokines through activation of NF-κB, and endoplasmic reticulum stress in the hypothalamus of rodents.^91, 92^ It is known that activation of this pathway disrupts the mechanisms linked to satiety and hunger, in part to those indicated in the arcuate nucleus that coordinate orexigenic and anorexigenic signals.^90, 91, 92^ In particular, the TLR-IKKβ-NF-κB pathway seems to be a molecular center that induces activation of additional proteins, among which the protein **TANK-BINDING KINASE 1** (TBK1) stands out. TBK1 is a member of the family of IKK proteins that are involved in the innate immune response, integrating multiple signals induced by viral infections, modulate the regulatory factor of interferon 3 (IRF3) and has been linked with a variety of autoimmune diseases and cancer.^93^ TBK1 activates the NF-κB complex by activating IKKβ.^94^ Initial reports has shown that TBK1 induces phosphorylation of the serine residue 994 of the insulin receptor, which correlates with insulin resistance *in vivo* and *in vitro* model of metabolic compromise.^95^ Recent evidence indicates that TBK1 may be a favorable drug target since inhibition of the molecular binomial TBK1/IKK-ɛ prevents generation of type 2 diabetes mellitus in obese mice.^96^ In this context, our research group has demonstrated that stimulation with saturated lipids favorably activates TBK1 who translocates to the region of lipids rafts in neurons of the hypothalamus of obese mice, which correlates with insulin resistance.^97^ Of interest, the unpublished data from our laboratory show that TBK1 is activated in microglia and correlates with the release of proinflammatory cytokines such as TNF-alpha and IL-6. Our data correlate with a recent study showing that intrahypothalamic inhibition of IKKɛ decreases TNF-alpha levels, improves insulin and leptin sensitivity, and decreases food intake.^98^ The molecular binomial TBK1/IKKɛ seems to have more versatile functions related to the regulation of body glucose homeostasis. In a recent study, it was shown that inhibition of TBK1/IKKɛ in obese mice induces the release of IL-6, which stimulates phosphorylation of the Stat3 pathway in the liver, suppressing gluconeogenesis.^99^

Overall, it is proposed that alterations in the control of glucose levels and food intake may be linked to the activation of microglia in the CNS through the TLR-IKKβ-NF-κB pathway.

## Central modulation of inflammation and microglia activation improve metabolic failure and cognitive impairment

Central modulation of inflammation during positive energy balance seems to improve insulin resistance and metabolic failure. Subcutaneous administration of liraglutide or canagliflozin, a sodium-glucose cotransporter 2 (SGLT2) inhibitor, to obese insulin-resistance mice blocks microglia activation in hypothalamus, which correlates with the recovery of insulin and glucose homeostasis, and decrease of fat and triglyceride content.^100, 101^ Also, the intracerebroventricular administration of the IKKβ/NF-κB blocker, prevents hypothalamic inflamation and improves body weight, fat accumulation and glucose, and energy homeostasis in an diet-induced obese model.^85, 102, 103^ In addition to the pharmacological modulation of inflammation in the CNS, physical activity might decreases central inflammation. Exercise decreases hypothalamic inflammation linked to IKKbeta pathway and improves insulin and leptin sensitivity and cognitive impairment in an obese rodent model.^104^

## Conclusions

The lipotoxicity associated with obesity generates metabolic disorders related to impaired glucose homeostasis. The molecular pathways linked to this metabolic process propose central and peripheral inflammation as a common pathogenic mechanism of the various manifestations of the metabolic syndrome. Inflammation in the CNS seems to be earlier than peripheral derangements. The TBK1/IKKɛ -NF-κB is the most reported central inflammation pathway inducing the release of proinflammatory cytokines during positive energy balance, leading to generation of type 2 diabetes. We contemplate that in later years, the molecular routes, together with TBK1/IKKɛ-NF-κB, which contribute to the regulation of the activation of NF-κB during obesity, will be deciphered.^105^
